# Indication for ‘Over the Scope’ (OTS)-Clip vs. Covered Self-Expanding Metal Stent (cSEMS) Is Unequal in Upper Gastrointestinal Leakage: Results from a Retrospective Head-to-Head Comparison

**DOI:** 10.1371/journal.pone.0117483

**Published:** 2015-01-28

**Authors:** Harald Farnik, Marlene Driller, Thomas Kratt, Carsten Schmidt, Martin Fähndrich, Natalie Filmann, Alfred Königsrainer, Andreas Stallmach, Michael Heike, Wolf O. Bechstein, Stefan Zeuzem, Jörg G. Albert

**Affiliations:** 1 Medizinische Klinik 1, Johann Wolfgang Goethe-Universität, Frankfurt, Germany; 2 Klinik für Allgemeine, Viszeral- und Transplantationschirurgie, Universitätsklinikum Tübingen, Tübingen, Germany; 3 Klinik für Innere Medizin IV, Klinikum der Universität Jena, Jena, Germany; 4 Medizinische Klinik Mitte Gastroenterologie, Städtisches Klinikum Dortmund, Dortmund, Germany; 5 Institut für Biostatistik und mathematische Modellierung, Universitätsklinikum Frankfurt, Frankfurt, Germany; 6 Klinik für Allgemein- und Viszeralchirurgie, Johann Wolfgang Goethe-Universität, Frankfurt, Germany; Hokkaido University, JAPAN

## Abstract

**Background:**

Intestinal perforation or leakage increases morbidity and mortality of surgical and endoscopic interventions. We identified criteria for use of full-covered, extractable self-expanding metal stents (cSEMS) vs. ‘Over the scope’-clips (OTSC) for leak closure.

**Methods:**

Patients who underwent endoscopic treatment for postoperative leakage, endoscopic perforation, or spontaneous rupture of the upper gastrointestinal tract between 2006 and 2013 were identified at four tertiary endoscopic centers. Technical success, outcome (e.g. duration of hospitalization, in-hospital mortality), and complications were assessed and analyzed with respect to etiology, size and location of leakage.

**Results:**

Of 106 patients (male: 75 (71%), female: 31 (29%); age (mean ± SD): 62.5 ± 1.3 years, 72 (69%) were treated by cSEMS and 34 (31%) by OTSC. For cSEMS vs. OTSC, mean treatment duration was 41.1 vs. 25 days, p<0.001, leakage size 10 (1-50) vs. 5 (1-30) mm (median (range)), and complications were observed in 68% vs. 8.8%, p<0.001, respectively. Clinical success for primary interventional treatment was observed in 29/72 (40%) vs. 24/34 (70%, p = 0.006), and clinical success at the end of follow-up was 46/72 (64%) vs. 29/34 (85%) for patients treated by cSEMS vs. OTSC; p = 0.04.

**Conclusion:**

OTSC is preferred in small-sized lesions and in perforation caused by endoscopic interventions, cSEMS in patients with concomitant local infection or abscess. cSEMS is associated with a higher frequency of complications. Therefore, OTSC might be preferred if technically feasible. Indication criteria for cSEMS vs. OTSC vary and might impede design of randomized studies.

## Introduction

Post-interventional intestinal leakage is a main cause of iatrogenic morbidity and mortality in surgical and nonsurgical procedures. Upper gastrointestinal tract tumor resection and bariatric surgery are accompanied by postoperative leakage in a considerable number of patients: the complication rate was 17%, and the reoperation rate was 7% in a recent meta-analysis of bariatric surgery [1]. The rate of anastomotic leakage was 7 to 12% and reoperations were necessary in 11% to 14% after esophagectomy for esophageal cancer in another recent report [2]. As well, advanced therapeutic endoscopy, i.e. endoscopic mucosal resection (EMR) and endoscopic submucosal dissection (ESD) harbor an increased risk of intestinal perforation [3]. The traditional standard of care is surgical repair in these complications. However, surgery is accompanied by significant mortality and morbidity, prolongs the hospital stay and increases the costs [4]. Therefore, endoscopic management is gaining popularity and has become a valuable alternative in many selected cases, recently [5].

The covered, removable self-expanding metal stent (cSEMS) and the ‘over the scope’ clip (OTSC) are the preferred alternatives to surgery for closure of iatrogenic leakage, and cSEMS are in use for treatment of postoperative leaks for a couple of years now [6,7]. But, cSEMS may migrate in 20% to 54% in large series [8], with dislodgement severely hampering this therapeutic approach. More recently, a newly designed metallic clip surpasses the size of previous through-the-scope (TTS) clips: The OTSC measures >10 mm in diameter and has been successfully used for treatment of gastrointestinal bleeding, fistula, and perforations [9–12]. However, preference of cSEMS vs. OTSC in post-interventional intestinal leakage is ambiguous and criteria for selection of one over the other are not established.

Herein we present our results of a large retrospective multicenter study of treating upper gastrointestinal leakage by applying the OTS-clip vs. cSEMS with respect to preference of the device, indication, treatment results and outcome. We verified the hypothesis that indication for cSEMS vs. OTSC is unequal and that designing prospective studies for interventional treatment of upper gastrointestinal leakage must take these indication criteria strictly into consideration.

## Materials and Methods

### Patients

Patients who had undergone interventional treatment (cSEMS insertion or OTSC application) of upper intestinal leakages were enrolled. Clinical data and endoscopic findings were recorded according to the standard of the respective study center. The study was approved by the institutional review board (Ethikkommission) of the Johann Wolfgang Goethe-University Hospital (No. 248/12) (Prof. Dr. med. H. Bratzke, Stellv. Vorsitzender der Ethik Komission) and the review boards of the university hospital of Tübingen, Jena and the hospital of Dortmund. All participants provide their written informed consent for the procedure. The ethics committee approved the consent procedure.

Endoscopic procedures were performed under conscious sedation with propofol and/or midazolam by using standard medical video endoscopes. Technical success was defined as an optimal placement of a cSEMS or an OTSC as intended by the investigator who assessed achievement of the intervention at the end of the investigation. Clinical success was defined as a demission from the hospital with resolution of symptoms that had been caused by the leakage. Complications were recorded at the hospital stay and during follow-up, e.g. coating defects, migration of cSEMS, dislocation of OTSC; ulceration, or bleeding in cSEMS vs. OTSC. Follow-up was determined time from day of treatment until last patient contact.

### The ‘over the scope’ clip (OTSC)

Application of the OTSC has previously been described [12]. In short, the OTSC system consists of a clip that is mounted on the tip of the endoscope by use of a transparent cap. The clip can be used together with the so-called ‘twin grasper’, i.e. a double grasping device for grabbing the two edges of a defect, or in combination with an anchoring device that helps to fix the tissue in fistulous disease and pull it into the transparent cap before releasing the clip. Once the damaged tissue is fully pulled into the applicator cap the OTS clip is deployed.

In this study, a-type and t-type OTSCs (11 mm, 12 mm, and 14 mm) were applied and twin type graspers and/or anchor devices were used according to the request of the investigator.

### The covered self-expanding metal stent (cSEMS)

All self-expanding metal stents (SEMS) were intended to be later removed and therefore, full covered SEMS (cSEMS), partially covered SEMS (pcSEMS) with bare ends and the self-expanding plastic stent were applied. cSEMS were placed over-the-wire (OTW) and under fluoroscopic and endoscopic guidance by experienced endoscopists using a medical endoscope with a working channel of 3.7 or 2.8 mm, respectively, for defining and controlling optimal positioning of the cSEMS.

### Evaluation of the treatment

OTSC and cSEMS were placed under endoscopic surveillance in all cases, and use of x-ray radiography was optional. Choosing cSEMS vs. OTSC was at the discretion of the investigator. In the majority of patients x-ray imaging or computed tomography was applied at the time of placement of the device and/or at follow-up studies. Abdominal computed tomography using an oral water-soluble contrast agent was performed before the procedure and subsequently according to the clinical course of the patients to exclude extra-intestinal fluid collections and abscess.

Treatment of the patients was evaluated from detecting the intestinal leak until last contact to the patient or until death. During clinical follow-up of the patient, either healing of the leak, death or surgical treatment was observed, or a second interventional treatment attempt was undergone in case that the first OTSC or cSEMS did not achieve complete sealing of the leakage. For interventional treatment, the investigator again chose either cSEMS or OTSC at his discretion. On a daily basis, treatment alternatives were discussed amongst the attending surgeons and interventionalists and alternative treatment approaches (e.g., surgical revision) taken into consideration. Independent from all closure techniques used, percutaneous drainage was placed in all patients with visible abscess in imaging techniques (e.g. computed tomography).

Failure of interventional treatment was defined in case of cSEMS removal with persistent leakage or inadvertent detachment of the OTSC or death.

### Statistics

Data were analyzed using Bias 10.12 (Bias for Windows), Microsoft Excel 2010, and WinSTAT for Microsoft Excel, Version 2012.1, Germany. Wilcoxon-Mann-Whitney-test was used. Ordinal variables were analyzed using Chi square test, Fisher’s exact test or Mantel-Haenszel test as appropriate.

Time to failure of the first treatment was analyzed with a competing risk analysis. Overall event-free survival was analyzed via Cox regression. The Kaplan-Meier method was used to calculate the actuarial relapse-free survival probabilities after the first session of endoscopic therapy.

All tests were two-tailed and a p-value of less than 0.05 was considered as significant.

## Results and Discussion

**Fig 1:**
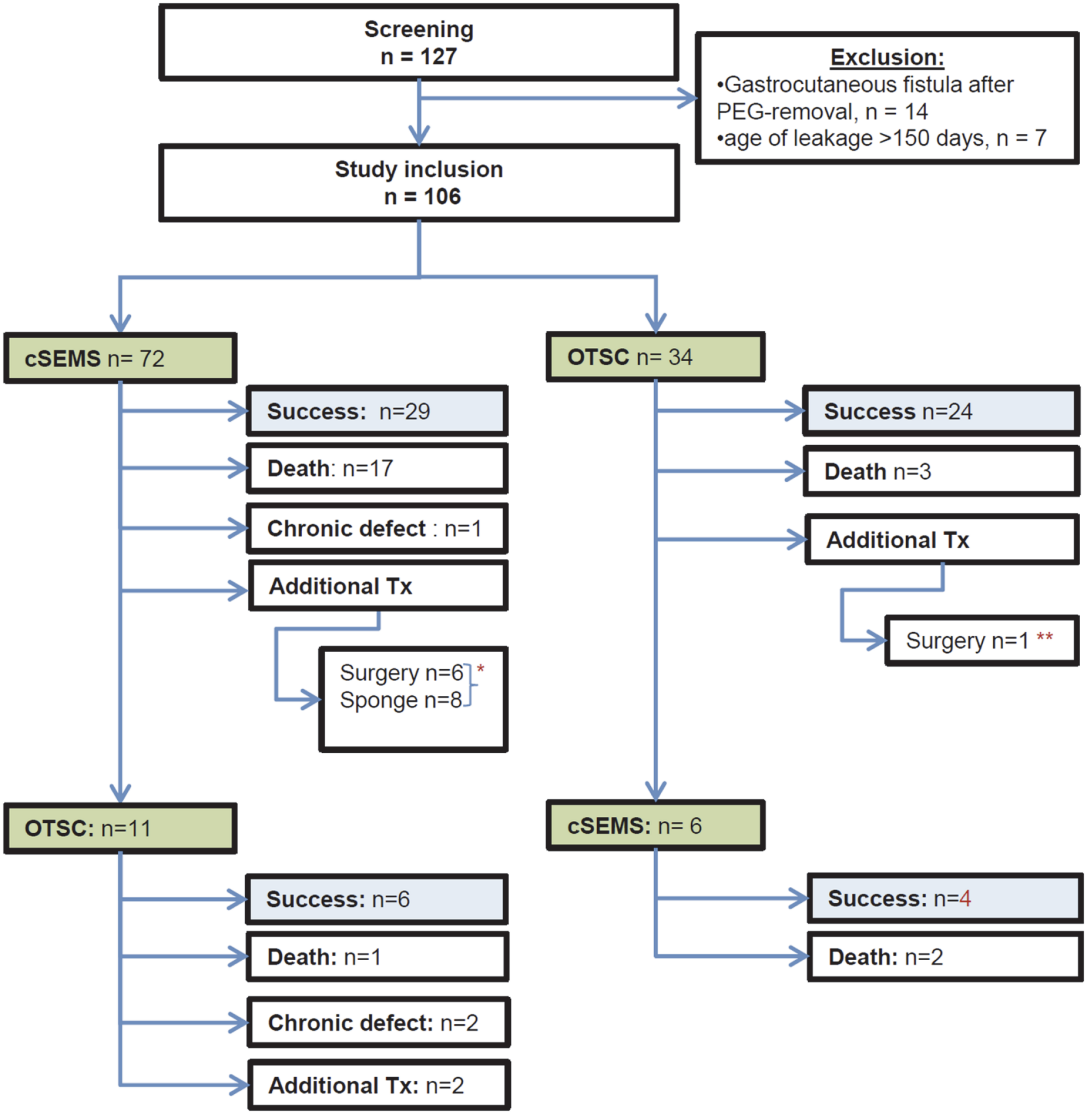
Workflow of the treatment of patients with upper gastrointestinal leakage. Success—clinical success defined as demission from the hospital with resolution of symptoms that had been caused by the leakage; sponge—endoscopic vacuum therapy; *success: n = 5, death: n = 5, success by further treatment: n = 4; **success: n = 1.

There were 106 patients from four tertiary endoscopic centers enclosed who had been treated between 2006 and 2013. Leakage of the upper gastrointestinal tract was caused by surgical procedures in 73 (69%), interventional endoscopic procedures in 24 (23%) or were spontaneous ruptures in 9 (8%) (Table 1). Out of 106 patients 53 male (74%) and 19 female (26%) were treated with cSEMS and 22 male (65%) and 12 female (35%) were treated with OTSC. Mean age ± SD in the cSEMS group was 63.3 ±13.4 vs 60.82 ±14.9 in the OTSC group, p = 0.5, (Fig. 1). None of the patients were treated in the context of bariatric surgery.

**Table 1:** Localization of the defect in patients who were treated for leakage from postoperative or post-interventional leakage.

Cause of leakage	Localization of the defect	n (%)
Postoperative leakage		73 (68.8%)
	Esophagus	5 (4.7%)
	Stomach	11 (10.4%)
	Duodenum	2 (1.9%)
	Esophago-gastric anastomosis	29 (27.4%)
	Esophago-jejunal anastomosis	23 (21.7%)
	Gastro-jejunal anastomosis	3 (2.8%)
Post-interventional leakage		24 (22.6%)
	Esophagus	12 (11.3%)
	Stomach	10 (9.4%)
	Duodenum	1 (0.9%)
	Jejunum	1 (0.9%)
Spontaneous rupture		9 (8.5%)
	Esophagus	8 (7.6%)
	Duodenum	1 (0.9%)

Out of 106 patients, 41 (39%) patients were treated in Tübingen, 27 (25%) in Frankfurt, 22 (21%) in Jena and 16 (15%) in Dortmund. Each center was free to choose its procedure in patients with postoperative or post-interventional leakage. However, we identified similar therapeutic approaches in all centers: In the first place, interdisciplinary (surgeon, interventional endoscopist) consent was obtained whether to suggest a surgical revision/operation or an interventional approach in a specific situation and the therapeutic approach was accordingly discussed with the patient. In case of a decision for an interventional treatment, all centers choose either cSEMS or OTSC as their first treatment option, choosing one or the other depending on the characterization of the lesion. At treatment failure, again all centers choose either cSEMS or OTSC or other treatment options (e.g. vacuum sponge therapy, others).

All centers have had extensive experience with both, OTSC and cSEMS before starting the study.

OTSCs were applied in 34 (32.1%) and cSEMS were inserted in 72 patients (67.9%) after diagnosing a leak in these patients, (Table 2). Etiology of leakage was a postoperative defect in 57 (79%) patients treated by cSEMS vs. 16 (47%) patients who had an OTSC placed (p<0.01), post-interventional in 9 (13%; cSEMS) vs. 15 (44%; OTSC, p<0.01), and a spontaneous rupture in 6 (8%; cSEMS) vs. 3 (9%; OTSC, p = n.s.), respectively. Seven (9%) patients who had cSEMS inserted and two (6%) patients in the OTSC group had undergone unsuccessful surgical revision before endoscopic treatment was initiated (p = 0.7). Out of 72 patients with cSEMS, 55 (76%) had inserted a full covered self-expanding metal stent, 8 (11%) had a partial covered self-expanding metal stent, 7 (10%) had a full and a partial covered stent combined and 2 (3%) had the Polyflex and a full covered self-expandable metal stent placed. 26 (76%) patients in the OTSC group were treated with a single clip and 8 (24%) were treated with simultaneous placement of two clips. Median number (range) of endoscopic procedures for insertion, replacement and/or removal of cSEMS vs. OTSC was 2 (1–10) vs. 1 (1–2), p<0.001, respectively.

**Table 2:** Patients who underwent treatment of upper gastrointestinal leakage with regard to cause and localization of the defect.

	cSEMS	OTSC	p
N	72	34	
Gender (m/f)	53 (73.6%) /19 (26.4%)	22 (64.7%) /12 (35.3%)	n.s.
Age (mean ± SD, years)	63.3 ± 13.4	60.8 ± 14.8	n.s.
**Cause of the intestinal leak**			
Postoperative; n (%)	57 (79%)	16 (47%)	<0.01
Post-interventional	9 (13%)	15 (44%)	<0.01
Spontaneous	6 (8%)	3 (9%)	n.s.
**Localization of the defect**			
Esophagus	18 (25%)	7 (20.6%)	n.s.
Esophago-gastric anastomosis	28 (38.9%)	1 (2.9%)	<0.01
Esophago-jejunal anastomosis	15 (20.8%)	8 (23.5%)	n.s.
Gastro-jejunal anastomosis	3 (4.2%)	0	
Stomach	7 (9.7%)	14 (41.2%)	<0.01
Duodenum	1 (1.4%)	3 (8.8%)	n.s.
Jejunum	0	1 (2.9%)	n.s.
**Age of the defect**			
<2d	14 (19%)	18 (53%)	
2–7d	28 (39%)	6 (18%)	
>7d	30 (42%)	10 (29%)	

n.s.—not statistically significant; SD—standard deviation.

### Success of treatment

Technical success was achieved for the patients treated by OTSC in 33/34 (97.1%) and in 71/72 (98.6%) for the cSEMS; p = n.s. For cSEMS vs. OTSC, median (range) treatment duration was 24 (0–356) vs. 8 (0–321) days, p<0.001. At initiating treatment, median (range) age of the leakage was 6 (0–50) vs. 1 (0–139) days for cSEMS vs. OTSC, p = 0.014. Median stay at hospital (mean, range) was 8 (19.4, 0–77) vs. 42 (53.5, 0–224) days for OTSC vs. cSEMS, p<0.001. Mean size of the leakage (median, range) was 12.6 (10, 1–50) vs. 7.1 (5, 1–30) mm for patients treated by cSEMS vs. OTSC, p<0.01. Complications (e.g. coating defects, migration of cSEMS; dislocation of OTSC; ulceration, bleeding in cSEMS or OTSC) occurred in 68% vs. 8.8% (p<0.001) for cSEMS vs. OTSC, respectively (Table 3).

**Table 3:** Performance of interventional treatment for upper gastrointestinal leakage in terms of a sealing attempt by OTSC vs. cSEMS.

		cSEMS	OTSC	p
n		72	34	
Technical success, n (%)		71 (98.6)	33 (97.1)	n.s.
Age of leakage (days)		7.9	10.9	n.s.
Size of leakage (mean in mm, median, range)		12.6 (10, 1–50)	7.1 (5, 1–30)	<0.01
Local infection/abscess, n (%)		51 (71)	15 (44)	<0.05
Treatment duration (days) median (range)		24 (0–356)	8 (0–321)	<0.05
Days on intensive care unit median (range)		7 (0–125)	0 (0–49)	<0.05
Rate of device migration/dislocation, n (%)		17 (24)	1 (3)	<0.01
Clinical success of primary treatment attempt, n (%)		29 (40)	24 (70)	

Clinical success in terms of age of the leakage, n (%)				
	<2d	6/14 (43)	16/18 (89)	
	2–7d	11/28 (39)	2/6 (33)	
	>7d	12/30 (40)	6/10 (60)	

Clinical success in terms of cause of the leakage, n (%)				
	Post-surgical	22/57 (39)	9/16 (56)	
	Post-interventional	4/9 (44)	13/15 (87)	
	Spontaneous	3/6 (50)	2/3 (66)	

Clinical success at the end of follow-up, n (%)		46 (64)	29 (85)	
Mortality at the end of follow-up, n (%)		23	5	n.s.

Clinical treatment success was observed in 29/72 (40%) vs. 24/34 (71%) patients in the cSEMS vs. OTSC group, p = 0.006, for the first attempt to heal the leakage by placing either cSEMS or OTSC (for final outcome see below).

Treatment failed in 36/72 (50%) patients in whom a cSEMS was inserted for interventional treatment. Ten patients died with death associated to the leakage, seven patients died from other reason; 25 patients underwent an additional treatment, while six patients underwent surgical revision (success n = 2; mortality n = 4), endoscopic vacuum therapy (8; success: 3; mortality: 1), or OTSC placement (11). One patient was demised from hospital with a persistent impairment due to the leak; Fig. 1. Treatment succeeded in 24/34 (70%) patients in case that OTSC had been chosen as an interventional treatment. One patient died with death associated to the leakage, two patients died from other reasons. Six patients were further treated by placement of a cSEMS.

**Fig 2:**
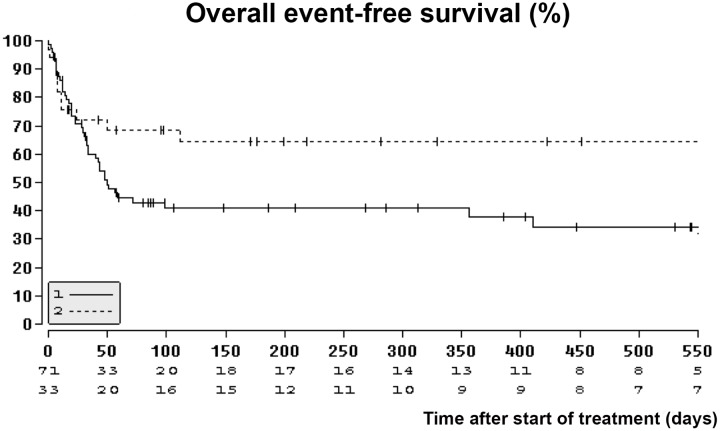
Kaplan-Meier curve with calculated actuarial event free survival probabilities after the first session of endoscopic therapy for the cSEMS group (solid line) and OTSC group (scattered line).

Thus, Patients who were managed by inserting a cSEMS at starting the treatment were observed to have a higher risk of treatment failure or death in comparison to those treated by OTSC (p = 0.035), Fig. 2. Median event-free survival was 50 days (95%-CI [34.6; 65.4]) in the cSEMS-group and greater than 50 days in the OTSC-group. Neither size nor age of the leak were significantly correlated with death or treatment failure. A parameter independently associated with death or treatment failure was postoperative vs. post-interventional leakage (HR: 2.59, 95% CI: 1.25–5.36; p = 0.031). Time to failure of the first treatment was analyzed with a competing risk analysis to account for the competing risk of death. The hazard of failure in the cSEMS—group was significantly higher than in the OTSC group (HR: 2.13, 95% CI: 1.01–4.49; p = 0.048).

### Further treatment

In case that the first interventional approach to treat the leak failed and that the leakage persisted after removal of the cSEMS or the defect recurred or persisted despite OTSC placement, the next treatment attempt was initiated dependent on the recommendation of the interdisciplinary team attending the patient. Additional treatment approaches included surgical revision and endoscopic vacuum therapy as depicted in Fig. 1, but in all other patients, a sealing attempt was again offered to the patient by use of cSEMS or OTSC and the device was selected at the discretion of the investigator depending on the morphology of the lesion.

In the group of patients that initially underwent cSEMS placement there were five male and six female patients treated by OTSC with a mean (± SD) age of 62.6 ± 20 years; Fig. 1. In the patient group that initially underwent OTSC placement, there were six male (no female) treated with cSEMS with a mean (± SD) age of 65.7 ± 8.6 years. The localization of the leakage was in the esophagus in one patient in the cSEMS group and in five in the OTSC group; at the esophago-gastric anastomosis in one (cSEMS) vs. two (OTSC); at the esophago-jejunal anastomosis in four (cSEMS) vs. two (OTSC), and in the stomach in none (cSEMS) vs. two (OTSC). Cause of the leakage was postoperative in six vs. seven patients (cSEMS vs. OTSC), post-interventional in none vs. three, and spontaneous rupture in none vs. one, respectively.

Technical success of OTSC placement for this second treatment approach was 9/11 vs. 6/6 (cSEMS). For cSEMS vs. OTSC, median (range) treatment duration was 82 (25–170) vs. 11 (0–244) days, and complications were observed in 4/11 vs. 2/6 patients, respectively. Mean age of the leak (median, range) at initiating this second treatment attempt was 16.7 (14, 10–356) vs. 72.6 (44, 1–30) days for cSEMS vs. OTSC, respectively. Clinical success of this second treatment approach was 4/6 vs. 6/11 for cSEMS vs. OTSC, respectively.

### Follow up

Patients were followed a mean of 267 (IQR: 31–373) days. From 106 patients, 75 (70.8%) were dismissed from the hospital with complete recovery from the intestinal leak. In-hospital mortality was 26.4% (28/106), and three patients were dismissed with a chronic defect. Successful healing of the leakage was observed in 46/72 (64%) patients initially treated by cSEMS and in 29/34 (85%) with application of OTSC; p<0.05. During the follow-up period, 23/72 (32%) patients died in the cSEMS group vs. 5/34 (14.7%) patients in the OTSC group.

### Discussion

Leakage of the upper gastrointestinal tract resulting from surgical or endoscopic interventions is a severe burden to the patient. Reliable and safe closure of perforations and leaks is the prior treatment goal. Removable cSEMS and OTSC are recent innovations that provide minimal-invasive closure. Both have become a major treatment option and are preferred over more invasive surgery for many patients who suffer from intestinal leakage [7,19] However, criteria for preference of cSEMS vs. OTSC in a particular patient are not well established and current case series are hampered by small patient numbers [20,12,21], Table 4. Other non-surgical treatment approaches, i.e. the use of metal clips, loops, rubber bands, fibrin glue, endoscopic vacuum therapy, and other techniques have been mainly replaced by cSEMS and OTSC in most cases [13–18].

**Table 4:** Clinical data on the use of the ‘over the scope’ (OTS) clip for closure of intestinal leakage.

Author	Year	Reference	n	Indication	Success rate
Kirschniak	2008	[9]	11	Bleeding / Perforation	100%
Repici	2009	[19]	9	Bleeding / Perforation	100%
Albert	2010	[12]	19	Perforation/Leakage	67%
Parodi	2010	[24]	10	Leakage and postoperative leakage	80%
Sandmann	2010	[25]	10	Postoperative leakage	90%
Seebach	2010	[26]	7	Perforation and postoperative leakage	85%
Von Renteln	2010	[27]	4	Fistula	50%
Kirschniak	2011	[20]	19	Postoperative leakage (n = 11), fistula (8)	100% (leakage), 38% (fistula)
Hagel	2012	[23]	17	Postoperative leakage	64.7%
Manta	2012	[11]	12	Postoperative leakage	92%
Mennigen	2013	[28]	14	Postoperative leakage	79%
Nishiyama	2013	[22]	23	Bleeding / Perforation	82.6%
Mönkemüller	2013	[29]	16	Postoperative leakage and fistula	75%
Own data	2014		34	Post-operative, post-interventional or spontaneous perforation	85%

In this study, we observed equal technical success rate of >95% for both, cSEMS and OTSC. However, indication for cSEMS and OTSC seem unequal. The leak was larger in the cSEMS than in the OTSC group (12.6 vs. 7.1 mm), mean treatment duration was longer for cSEMS (45.6 vs. 19.8 days), and hospitalization was longer (45.6 vs. 19.8 days). Moreover, local inflammation/abscess was more frequently accompanying leakage in the cSEMS group (71%) than in the OTSC group (44%). Primary clinical success was observed in 40% vs. 71% of patients treated by cSEMS vs. OTSC (p = 0.006) at the initial attempt to treat the leakage and in 64% vs. 85% (p = 0.04) at final outcome including additional therapies in case that the initial treatment had failed. We are convinced that the difference in clinical outcome might not so much be due to the device itself—i.e. cSEMS or OTSC—, but rather attribute to the different comorbidities of the patients and the disparity of the lesion itself.

We thereby confirm some of the findings of smaller case series: A large lesion size (greater than 20 mm) and a delayed diagnosis (more than 1 week) were the major contributing factors for an unsuccessful treatment by OTSC in one study [22]. Immediate closure of iatrogenic perforations of the upper and lower GI tract was successful in all cases in another study, but a permanent closure of fistulas could not be achieved in all cases with the OTSC clip [20]. Moreover, size <10mm and vital margins of perforations were predictive for treatment success of OTSC [23].

In our study, complications occurred more often after placing cSEMS (66.7% vs. 5.9% in OTSC), i.e. the stents dislodged in a significant number of cases (24%) but only rarely OTS clips were loosening from its position (3%). Stent migration is a major reason of treatment failure in cSEMS therapies and may occur in up to 54% in large series [8].

## Conclusions

cSEMS and OTSC are rather complementary means than to be mutually exchangeable. OTSC are highly effective, and good outcome and low complication profile suggest that the OTSC should be the first choice in all cases when it is technically feasible and the diameter of the lesion is not too large to grasp it. In patients with larger defects and already infection accompanying the leak, and in case that prolonged treatment is anticipated, cSEMS placement might be preferred. In addition, difference of indication criteria for cSEMS vs. OTSC might impede designing randomized studies. Clear definition of inclusion criteria at randomized studies should rely on our findings.
